# Early effects of sodium-glucose co-transporter 2 inhibitors in type 2 diabetes: study based on continuous glucose monitoring

**DOI:** 10.1186/s13098-017-0258-5

**Published:** 2017-08-04

**Authors:** Keiichi Torimoto, Yosuke Okada, Kenji Koikawa, Yoshiya Tanaka

**Affiliations:** First Department of Internal Medicine, School of Medicine, University of Occupational and Environmental Health, 1-1 Iseigaoka, Yahatanishi-ku, Kitakyushu, 807-8555 Japan

**Keywords:** Sodium-glucose co-transporter 2 inhibitor, Type 2 diabetes mellitus, Continuous glucose monitoring

## Abstract

**Background:**

Inhibitors of sodium-glucose co-transporter 2 (SGLT2) have immediate glucose-lowering effects by promoting urinary glucose excretion, without altering insulin level. Only a few studies have evaluated blood glucose dynamics in the early period after administration. The present retrospective study was designed to determine the immediate effects of SGLT2 inhibitors on blood glucose dynamics.

**Methods:**

The study subjects were 24 patients with type 2 diabetes whose blood glucose dynamics were evaluated with continuous glucose monitoring for 1 week before and after initiation of SGLT2 inhibitor therapy. Blood glucose dynamics were examined on days −1, 0 (treatment commencement day), 3, and 7 by evaluating different continuous glucose monitoring parameters and blood glucose before each meal. Furthermore, blood glucose levels at 1 and 2 h after each meal and daily urinary glucose levels were determined.

**Results:**

A significant reduction in blood glucose levels 2 h after breakfast was observed between the day before treatment (249.8 mg/dL) and the day treatment started (218.9 mg/dL). The mean daily blood glucose level improved significantly from 201.4 to 142.3 mg/dL from the day the treatment started. Blood glucose variation also improved significantly by week 1, as demonstrated by changes in standard deviation and mean amplitude of glycemic excursions (from 39.6 to 31.7 and 106.9 to 87.4 mg/dL, respectively). The percent time at blood glucose <70 mg/dL remained unchanged while urinary glucose on day 7 correlated with minimum blood glucose level (r = 0.474, p = 0.022).

**Conclusions:**

The results showed that the SGLT2 inhibitors lower blood glucose from 2 h after the first dose and improve blood glucose variation by week 1 after start of the treatment. Furthermore, SGLT2 inhibitors did not alter the incidence of hypoglycemic episodes at week 1, suggesting that SGLT2 protects against severe hypoglycemia by inhibiting urinary glucose excretion.

## Background

Sodium-glucose co-transporter 2 (SGLT2) inhibitors, a new class of oral hypoglycemic drugs, are widely used clinically. These agents selectively inhibit SGLT2 activity and promote urinary glucose excretion to reduce blood glucose without targeting the main pathology of type 2 diabetes mellitus (T2DM), such as insulin resistance and insulin hyposecretion [1]. A meta-analysis of clinical trials showed that the drug was effective in improving glycated hemoglobin (HbA1c) levels, with a mean change of −0.66%. The drug also reduced body weight, with a mean change of −1.80 kg, and improved systolic blood pressure, with a mean change of −4.45 mmHg [2]. Other studies of 24-h blood glucose dynamics using continuous glucose monitoring (CGM) reported that SGLT2 inhibitors improve mean blood glucose (MBG) level, postprandial hyperglycemia, and variation in blood glucose level within a few weeks [3–5].

SGLT2 inhibitors seem to exert their therapeutic effects early since they are known to promote urinary glucose excretion to correct hyperglycemia without directly influencing insulin secretory capacity or sensitivity. However, few studies have closely examined blood glucose dynamics soon after the start of treatment. The early effect of SGLT2 inhibitors on blood glucose dynamics including time of onset of action remains unknown.

The present study was designed to evaluate blood glucose dynamics immediately after the start of SGLT2 inhibitor therapy (i.e., the immediate effect on blood glucose level), time of onset of action, and incidence of hypoglycemia in patients with T2DM admitted to our hospital.

## Methods

### Subjects

Among the T2DM patients admitted to the University of Occupational and Environmental Health, Japan between May 2014 and June 2016, who were being treated with an SGLT2 inhibitor, we selected 24 subjects who had undergone 1-week evaluation of blood glucose dynamics using a CGM system (CGMS^®^ Gold™ or iPro2, Medtronic MinMed, Inc.). Other inclusion criteria, used in this study, are the following: (1) age >20 years; (2) blood glucose level at admission <300 mg/dL, (3) absence of diabetic ketosis or non-ketotic hyperosmolar coma, and (4) absence of cardiac arrhythmias. Patients with aspartate aminotransferase (AST) ≥100 IU/L or alanine aminotransferase (ALT) ≥100 IU/L, patients with serum creatinine level ≥2.0 mg/dL or estimated glomerular filtration rate (eGFR) <30 mL/min/1.73 m^2^, infectious diseases, acute coronary syndrome, anemia and/or using erythropoiesis stimulating agents were also excluded from the study.

Blood glucose dynamics were evaluated on day −1, 0 (the day of commencement of treatment), 3, and 7. The primary endpoint was the time to onset of action of SGLT2 inhibitors.

The study protocol was approved by the ethics review committees of the University of Occupational and Environmental Health and the participating medical centers. Informed consent was obtained from all subjects.

### The CGM system

A previous study indicated that interstitial glucose concentrations measured by CGM correlate with venous blood glucose levels [6]. CGM measurements represent glucose concentrations in the interstitial fluid, but since the introduction of the self-monitoring blood glucose (SMBG) technique, the measured value is considered to represent blood glucose level. Because the iPro2 has insufficient stability within 24 h after its placement, the data after 24 h were used in order to avoid bias related to CGM placement or insufficient stability of the monitoring system. All patients received optimal meals (25 kcal/kg of ideal body weight; 60% carbohydrate, 15–20% protein and 20–25% fat) during CGM. The data recorded by the CGM using a SMBG device were retrieved and the MBG, standard deviation (SD), coefficient of variation (CV), percentage time at glucose level <70, 70–140, and ≥140 mg/dL, mean amplitude of glycemic excursion (MAGE), largest amplitude of glycemic excursion (LAGE), M value, mean postprandial glucose excursion (MPPGE), maximum (Max) and minimum blood glucose level (Min) before each meal, and blood glucose at 1 and 2 h after each meal were computed. MAGE, which was proposed by Service et al. [7], represents fluctuations in blood glucose levels over a 24-h period and was calculated from the daily variations in blood glucose level. The M value is calculated as published previously [8]: ${{\Sigma \left| { 10 \times { \log }\left( {{\text{GR}}t/ 100} \right)} \right|^{ 3} } \mathord{\left/ {\vphantom {{\varSigma \left| { 10 \times { \log }\left( {{\text{GR}}t/ 100} \right)} \right|^{ 3} } n}} \right. \kern-0pt} n}$, where GRt is the glucose reading at time *t*, and *n* is the number of observations. The M value index is used to quantify the change in postprandial blood glucose, as proposed by Schlichtkrull et al. [9]. To assess postprandial glucose excursions from CGM data, MPPGE was calculated as the arithmetic mean of the differences between the postprandial peak glucose values and the corresponding preprandial glucose values for meals.

### Data handling and statistical analysis

All numerical values are expressed as mean ± SD, and their normal distribution was tested using the Shapiro–Wilk test. The change in each CGM parameter over time was analyzed using repeated measures analysis of variance, and any noted significant difference was subjected to multiple comparisons using Tukey’s test. The correlation between the change in urinary glucose and that in each CGM parameter was analyzed using the Pearson’s correlation if it was normally distributed; otherwise Spearman’s correlation was used. A difference with a p value of <0.05 was considered significant. The statistical package for the social sciences software version 22.0 was used for the data analysis (SPSS Inc., Chicago, IL).

## Results

### Baseline characteristics

The clinical characteristics of the 24 subjects (14 men and 10 women) are shown in Table 1. The mean age, duration of morbidity, and HbA1c level were 56.2 ± 8.9 years (range 40–71 years), 5.3 ± 6.8 years (range 0–25 years), and 10.1 ± 2.4% (range 7.3–17.0%), respectively.

**Table 1:** Clinical characteristics of study participants with type 2 diabetes (n = 24)

Age, years	56.2 ± 8.9
Gender, male/female	14/10
Body mass index, kg/m^2^	26.7 ± 4.2
Duration of diabetes, years	5.3 ± 6.8
Systolic blood pressure, mmHg	122.6 ± 12.0
Diastolic blood pressure, mmHg	76.3 ± 11.0
Diabetes-related complications	
Neuropathy, n (%)	9 (37.0)
Retinopathy, n (%)	7 (29.0)
Nephropathy, n (%)	6 (25.0)
Fasting glucose, mmol/L	177.0 ± 56.0
IRI, μU/mL	9.3 ± 6.4
HbA1c, %	10.1 ± 2.4
Urinary CPR, μg/day	109.9 ± 61.2
AST, U/L	26.0 ± 12.3
ALT, U/L	28.1 ± 14.1
eGFR, mL/min/1.73 m^2^	86.1 ± 18.5
Uric acid, mg/dL	5.3 ± 1.3

Data are mean ± SD or *n* (%)

*IRI* immunoreactive insulin, *HbA1c* glycated hemoglobin, *CPR* C peptide immunoreactivity, *eGFR* estimated glomerular filtration rate

### Pharmacotherapy at admission and discharge

All subjects were treated with one SGLT2 inhibitor in addition to dietary therapy once hospitalized. The SGLT2 inhibitors ipragliflozin, dapagliflozin, and tofogliflozin were used in 17, 4, and 2 subjects, respectively and canagliflozin in 1 subject. This study was conducted in clinical settings. The antidiabetic therapy was modified in 4 patients when adding SGLT2 inhibitors, considering the background and treatment goal for each patient: SU drug treatment was discontinued in 1 patient; metformin treatment was added in 1 patient; and treatment with a DPP4 inhibitor and insulin were discontinued in 1 patient each.

### Effects of treatment with SGLT2 inhibitor on CGM parameters

**Fig. 1:**
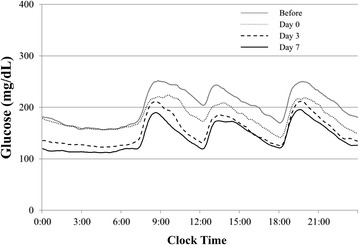
The average glucose level of the 24 patients measured by CGM. The average level was measured by CGM on day −1 (*gray line*), day 0 (*dotted line*), day 3 (*dashed line*) and day 7 (*solid line*) of treatment with SGLT2 inhibitor

Table 2 and Fig. 1 summarize the effects of treatment with SGLT2 inhibitors on various CGM parameters. The MBG and M value significantly decreased on day 0 and further decreased by day 7. The SD, MAGE, and LAGE improved significantly by day 7. The percentage time at ≥140 mg/dL, Max, and Min significantly decreased on day 3 and further improved by day 7 while the percentage time at <70 mg/dL and MPPGE remained unchanged.

**Table 2:** CGM parameters and urinary glucose level of 24 participants treated with SGLT2-I

CGM parameters	Before	Day 0	Day 3	Day 7	*p* value
MBG, mg/dL	201.4 ± 55.6	180.8 ± 43.2*	154.0 ± 29.1*	142.3 ± 20.2*	<0.001
SD, mg/dL	39.6 ± 13.5	34.7 ± 12.8	34.4 ± 12.8	31.7 ± 9.2*	0.006
CV, %	20.0 ± 5.5	19.1 ± 4.7	22.3 ± 6.7	22.6 ± 6.5	0.015
Time at >140 mg/dL, %	79.4 ± 24.7	74.7 ± 25.7	52.9 ± 25.6*	47.0 ± 23.7*	<0.001
Time at <70 mg/dL, %	0	0	0.2 ± 1.1	0.9 ± 3.6	0.355
Time at 70–140 mg/dL, %	20.6 ± 24.7	25.3 ± 25.7	46.8 ± 25.5*	52.1 ± 22.8*	<0.001
MAGE, mg/dL	106.9 ± 42.0	99.1 ± 38.8	96.3 ± 32.6	87.4 ± 25.2*	0.049
LAGE, mg/dL	147.5 ± 46.2	148.1 ± 51.0	132.9 ± 51.2	121.2 ± 38.3*	0.009
M value, mg/dL	47.7 ± 39.8	32.6 ± 27.1*	19.9 ± 15.2*	14.5 ± 6.7*	<0.001
MPPGE, mg/dL	80.8 ± 30.6	76.8 ± 34.9	85.5 ± 35.2	79.3 ± 23.4	0.472
Max, mg/dL	282.4 ± 64.8	271.4 ± 66.1	237.3 ± 53.0*	217.3 ± 31.4*	<0.001
Min, mg/dL	135.0 ± 43.9	123.3 ± 29.3	104.4 ± 19.4*	96.2 ± 22.1*	<0.001
BG_BB_, mg/dL	176.5 ± 51.0	170.5 ± 37.5	135.2 ± 30.5*	121.9 ± 18.9*	<0.001
BG_1hAB_, mg/dL	226.7 ± 56.7	207.4 ± 59.7	195.1 ± 38.9*	170.6 ± 28.6*	<0.001
BG_2hAB_, mg/dL	249.8 ± 60.0	218.9 ± 67.5*	204.5 ± 48.6*	183.1 ± 36.9*	<0.001
BG_BL_, mg/dL	206.5 ± 65.0	174.2 ± 60.6*	131.8 ± 39.8*	120.1 ± 25.6*	<0.001
BG_1hAL_, mg/dL	241.1 ± 78.1	202.9 ± 65.9*	179.3 ± 41.5*	169.1 ± 33.8*	<0.001
BG_2hAL_, mg/dL	229.8 ± 69.4	205.7 ± 67.6	182.5 ± 45.1*	171.4 ± 31.0*	<0.001
BG_BD_, mg/dL	169.2 ± 59.4	142.3 ± 36.7*	125.5 ± 28.8*	121.1 ± 28.0*	<0.001
BG_1hAD_, mg/dL	238.0 ± 68.0	204.6 ± 41.1*	192.7 ± 40.0*	183.1 ± 21.5*	<0.001
BG_2hAD_, mg/dL	248.4 ± 72.8	217.8 ± 58.0*	205.7 ± 45.4*	187.7 ± 33.3*	<0.001
Urinary glucose, g/day	18.8 ± 26.6		84.3 ± 31.9*	71.3 ± 31.7*	<0.001

Data are mean ± SD or n (%)

Repeated measures ANOVA was used to determine the association-treatment difference

*CGM* continuous glucose monitoring, *SGLT2*-*I* sodium glucose cotransporter 2 inhibitor, *MBG* mean blood glucose, *SD* standard deviation, *CV* coefficient of variation, *MAGE* mean amplitude of glycemic excursions, *LAGE* largest amplitude of glycemic excursions, *MPPGE* mean postprandial glucose excursions, *BG* blood glucose, *BB* before breakfast, *AB* after breakfast, *BL* before lunch, *AL* after lunch, *BD* before dinner, *AD* after dinner

* p < 0.05 for differences with the value at before treatment

We also examined the blood glucose profile on the day treatment was initiated. Analysis of the change in blood glucose from day −1 to 0 showed that the levels before and 1 h after breakfast remained the same (176.5–170.5 and 226.7–207.4 mg/dL, respectively) while those at 2 h after breakfast and before lunch significantly decreased (249.8–218.9 and 206.5–174.2 mg/dL, respectively).

### Correlation analysis of factors associated with urine glucose on day 7 of SGLT2 inhibitor treatment

Urinary glucose level increased significantly from day 3 (p < 0.001), with values at 18.8 ± 26.6, 84.3 ± 31.9, and 71.3 ± 31.7 g/day before the start of treatment, at day 3, and at day 7, respectively. The relationship between changes in CGM parameters and urinary glucose before treatment and by day 7 is shown in Table 3. The change in urinary glucose (Δurinary glucose) correlated with ΔSD (r = −0.468, p = 0.024), ΔCV (r = −0.654, p = 0.001), ΔMAGE (r = −0.520, p = 0.011), ΔLAGE (r = −0.465, p = 0.025), ΔMPPGE (r = −0.436, p = 0.038), and Min (r = 0.418, p = 0.047), but not with Δpercent time at ≥140, <70, and 70–140 mg/dL or the ΔM value.

**Table 3:** Analysis of factors correlated with urinary glucose changes at day 7 of SGLT2 inhibitor treatment

	ΔUrinary glucose	*p* value
ΔMBG	0.239	0.272
ΔSD	−0.468*	0.024
ΔCV	−0.654**	0.001
ΔTime at >140 mg/dL	0.352	0.100
ΔTime at <70 mg/dL	−0.277	0.201
ΔTime at 70–140 mg/dL	−0.314	0.144
ΔMAGE	−0.520*	0.011
ΔLAGE	−0.465*	0.025
ΔM value	0.103	0.640
ΔMPPGE	−0.436*	0.038
ΔMax	−0.027	0.904
ΔMin	0.418*	0.047

Data are results of Pearson correlation analysis of variables with normal distribution (ΔSD, ΔCV, ΔTime at >140 mg/dL, ΔTime at 70–140 mg/dL, ΔMAGE, ΔLAGE, ΔMMPGE and ΔMax) and Spearman rank correlation for variables with skewed distribution

*IRI* Immunoreactive insulin, *HbA1c* glycated hemoglobin, *CPR* C peptide immunoreactivity, *eGFR* estimated glomerular filtration rate, *CGM* continuous glucose monitoring, *SGLT2*-*I* sodium glucose cotransporter 2 inhibitor, *MBG* mean blood glucose, *SD* standard deviation, *CV* coefficient of variation, *MAGE* mean amplitude of glycemic excursions, *LAGE* largest amplitude of glycemic excursions, *MPPGE* mean postprandial glucose excursions, *BG* blood glucose, *BB* before breakfast, *AB* after breakfast, *BL* before lunch, *AL* after lunch, *BD* before dinner, *AD* after dinner

* p < 0.05, ** p < 0.01

Table 4 describes the relationship between CGM parameters and urinary glucose on day 7. Urinary glucose correlated with MBG (r = 0.414, p = 0.050), percent time at ≥140 (r = 0.468, p = 0.024) and 70–140 mg/dL (r = −0.470, p = 0.023), and Min (r = 0.474, p = 0.022), but not with SD, CV, percent time at <70 mg/dL, MAGE, LAGE, M value, MPPGE, or Max.

**Table 4:** Correlation analysis of the factors associated with urinary glucose on day 7 of treatment with SGLT2 inhibitors

	Urinary glucose	*p* value
MBG	0.414*	0.050
SD	−0.209	0.337
CV	−0.305	0.157
Time at >140 mg/dL	0.468*	0.024
Time at <70 mg/dL	−0.228	0.298
Time at 70–140 mg/dL	−0.470*	0.023
MAGE	0.135	0.539
LAGE	−0.381	0.073
M value	0.303	0.159
MPPGE	−0.093	0.673
Max	0.012	0.955
Min	0.474*	0.022

Data are results of Pearson correlation analysis of variables with normal distribution (MBG, CV and Max) and Spearman rank correlation for variables with skewed distribution

*IRI* immunoreactive insulin, *HbA1c* glycated hemoglobin, *CPR* C peptide immunoreactivity, *eGFR* estimated glomerular filtration rate, *CGM* continuous glucose monitoring, *SGLT2*-*I* sodium glucose cotransporter 2 inhibitor, *MBG* mean blood glucose, *SD* standard deviation, *CV* coefficient of variation, *MAGE* mean amplitude of glycemic excursions, *LAGE* largest amplitude of glycemic excursions, *MPPGE* mean postprandial glucose excursions, *BG* blood glucose, *BB* before breakfast, *AB* after breakfast, *BL* before lunch, *AL* after lunch, *BD* before dinner, *AD* after dinner

* p < 0.05

## Discussion

The present study followed 24 T2DM patients and investigated the effect of SGLT2 inhibitors on their blood glucose levels using CGM. Treatment with SGLT2 inhibitors (1) lowered glucose level from 2 h after administration of the first dose, (2) improved the variation in blood glucose level by 1 week after the commencement of treatment, and (3) did not increase the incidence of hypoglycemia after 1 week. However, urinary glucose excretion was more profoundly inhibited in subjects with lower minimum blood glucose levels than it was in those with higher levels. Few studies have evaluated blood glucose dynamics immediately after the start of treatment with SGLT2 inhibitors. In this study, we found that SGLT2 inhibitors induced an immediate decrease in glucose levels, reduced the variations in blood glucose levels, and regulated urinary glucose excretion to prevent hypoglycemia.

SGLT2 inhibitors specifically act on SGLT2 to inhibit tubular glucose reabsorption and promote glucose excretion in urine, thereby lowering blood glucose level [10]. Meta-analysis studies, investigating the long-term effects of SGLT2 inhibitors, have reported an improvement in blood glucose with HbA1c levels of 0.49–0.66% [2, 11, 12]. Another study on the early effect of SGLT2 inhibitors in Zucker diabetic fatty rats showed that canagliflozin inhibited renal glucose reabsorption from 2 h after administration and reduced blood glucose immediately after administration [13]. Studies in humans using CGM also reported that SGLT2 inhibitors improved MBG, postprandial blood glucose, and variation in blood glucose level within a few weeks [3–5]. Although few studies have closely examined the time of onset of effect, the present study showed that SGLT2 inhibitors lowered blood glucose level within 2 h of administration.

SGLT2 inhibitors suppress accelerated renal glucose reabsorption in patients with diabetes and promote urinary excretion of excessive blood glucose to correct hyperglycemia [10]. Furthermore, SGLT2 inhibitors reduce the chance of glucotoxicity because they induce urinary glucose excretion and thereby reduce blood glucose without changes in insulin level [14, 15]. In fact, earlier studies reported that SGLT2 inhibitors neutralized glucotoxicity to improve insulin secretory capacity [16] and resistance [17]. The present study showed that SGLT2 inhibitors improved the variation in blood glucose level within 1 week, and that urinary glucose excretion correlated with indices of blood glucose variation. These results suggest that correction of hyperglycemia through enhanced urinary glucose excretion may improve glucotoxicity, contributing to improvement in blood glucose variation.

In the present study, neither the incidence nor duration of hypoglycemia increased 1 week after the start of treatment. SGLT2 inhibitors have been reported to promote glucagon production [18, 19], which may limit the likelihood of development of hypoglycemia. Although glucagon was not measured in the present study, the Min level at the 1 week time point correlated with urinary glucose, suggesting that urinary glucose excretion is more profoundly inhibited in patients with lower minimum blood glucose levels than it was in those with higher levels. SGLT2 inhibitors are unlikely to pose a risk of inducing hypoglycemia because they do not stimulate insulin secretion or inhibit counter-regulatory response during hypoglycemia. Furthermore, hypoglycemia is unlikely to occur because glucose reabsorption accelerates following activation of SGLT1 [20] and gluconeogenesis is enhanced following a fall in blood glucose level [18, 19]. In addition, the effect of SGLT2 inhibitors on urinary glucose excretion is considered to be dependent on the rate of glucose filtration into primary urine. That is, urinary glucose excretion is expected to be suppressed when blood glucose level is lowered below the threshold of glucose reabsorption in the renal tubules [1], and thus a further decrease in blood glucose level is prevented. In accordance with this, our results suggest the presence of a threshold value for the minimal blood glucose level.

Studies on patients receiving high/low carbohydrate diets [21] and high/low glycemic index diets [22] reported that SGLT2 inhibitors were equally effective in improving glycemic control and increasing urinary glucose excretion, regardless of the condition. Therefore, we consider the results of the present study to be applicable to patients consuming meals of different composition. In this study, we used 4 SGLT2 inhibitors, and there were no differences in efficacy among the different drugs. However, the blocking rates are reported to be different among SGLT2 inhibitors [23]. Thus, the effects of glucose reduction and urinary glucose excretion may be different among the drugs. Future studies should compare effects among the different SGLT2 inhibitors.

The present study has several limitations. First, it was retrospective in nature. Second, all subjects were hospitalized and, therefore, the applied strict dietary therapy may have modified the effect of the SGLT2 inhibitors. Third, because glucagon level s were not measured, the mechanism by which SGLT2 inhibitors suppress the likely development of hypoglycemia was not sufficiently examined. Fourth, the sample size was small, and the population was a biased. The subjects of the present study were a poorly-controlled diabetic population with a mean HbA1c of 10.1%. Thus, the glucose-lowering effect may not be evident and the change in urinary glucose excretion rate may be different when the drug is administered to a better-controlled diabetic population. Fifth, the effect of SGLT2 inhibitors probably continues beyond the study period of 1 week. Therefore, a long-term study is needed in the future.

## Conclusions

The present study showed that SGLT2 inhibitors lowered blood glucose levels from 2 h after administration and improved the variation in blood glucose level within 1 week of treatment. The incidence of hypoglycemia was not increased at 1 week after the start of the treatment, suggesting that inhibiting urinary glucose excretion prevents lowering of the Min level. Therefore, treatment with SGLT2 inhibitors is beneficial and safe and should be introduced as a viable treatment option, based on its immediate glucose-lowering effect.

## Abbreviations

SGLT2: sodium-glucose co-transporter 2
T2DM: type 2 diabetes mellitus
HbA1c: glycated hemoglobin
CGM: continuous glucose monitoring
MBG: mean blood glucose
SMBG: self-monitoring blood glucose
SD: standard deviation
CV: coefficient of variation
MAGE: mean amplitude of glycemic excursion
LAGE: largest amplitude of glycemic excursion
MPPGE: mean postprandial glucose excursion
Max: maximum blood glucose level
Min: minimum blood glucose level
